# Mutual information based weighted variance approach for uncertainty quantification of climate projections

**DOI:** 10.1016/j.mex.2023.102063

**Published:** 2023-02-05

**Authors:** Archana Majhi, C.T. Dhanya, Sumedha Chakma

**Affiliations:** Department of Civil Engineering, Indian Institute of Technology Delhi, Hauz Khas, New Delhi, India

**Keywords:** Model independence, Mutual information, Independence weight, Uncertainty quantification, Mutual-information-based weighted variance approach for climatic projections uncertainty quantification

## Abstract

Future climate projections are a vital source of information that aid in deriving effective mitigation and adaptation measures. Due to the inherent uncertainty in these climate projections, quantification of uncertainty is essential for increasing its credibility in policymaking. While quantifying the uncertainty, often the possible dependency between the General Circulation Models (GCMs) due to their shared common model code, literature, ideas of representation processes, parameterization schemes, evaluation datasets etc., are ignored. As this will lead to wrong conclusions, the inter-model dependency and the respective independence weights need to be considered, for a realistic quantification of uncertainty. Here, we present the detailed step-wise methodology of a “mutual information based independence weight” framework, that accounts for the linear and nonlinear dependence between GCMs and the equitability property.

• A brief illustration of the utility of this method is provided by applying it to the multi-model ensemble of 20 GCMs.
• The weighted variance approach seemingly reduces the uncertainty about one GCM given the knowledge of another.

## Graphical abstract

**Figure:**
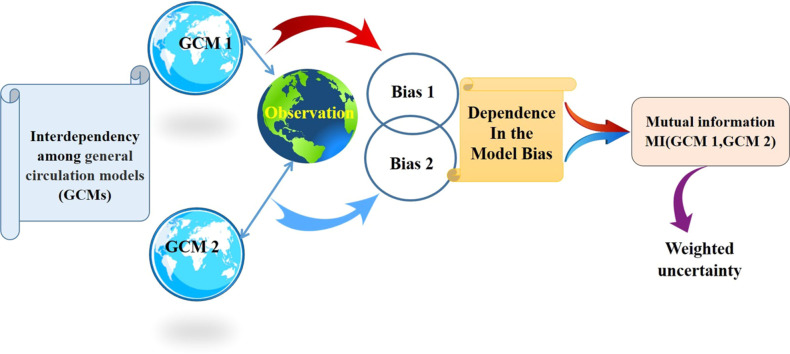

Specifications table

**Table:** 

Subject area:	Earth and Planetary Science
More specific subject area:	Climate change
Method Name:	Mutual-information-based weighted variance approach for climatic projections uncertainty quantification
Name and reference of original method:	Dionisio, A., Menezes, R., & Mendes, D. A. (2004). Mutual information: a measure of dependency for nonlinear time series. *Physica A: Statistical Mechanics and its Applications*, *344*(1–2), 326–329.
Resource availability:	For Model data (Precipitation 3-hourly) https://esgf-node.llnl.gov/search/cmip5/ For Observed data (Precipitation 3-hourly) https://rda.ucar.edu/datasets/ds628.0/

## Method details

### Background

The climate projections are inherently uncertain due to the uncertainty associated with different future scenarios, model structures, and initial conditions [1,2]. The long-term projections are not reliable, majorly due to the incomplete understanding of nature and its representation in climate models [3,4]. With the development in climate science, the climate models have improved resolution, representation of processes, a combination of socioeconomic development and GHG emission scenarios, and physical parameterization schemes (convection, cloud, radiation, land surface) [5,6]. Despite such improvement, model uncertainty is still a dominant source of uncertainty, and needs to be quantified. However, these climate models may be dependent on each other, as they may share common numerical schemes, parameterization processes, or even resolution [7], [8], [9], which in turn should be considered while quantifying the model uncertainty. To date, there is limited availability of transparent documents regarding model development [8,9]. The climate modeling groups use the approach of sharing ideas and code, to avoid duplication of efforts, and therefore, the extent of dependence needs to be measured [10,11].

Despite this, very few studies attempted to investigate the independence of climate models. The assumption that the same version models are dependent, as they share similar components [13], may be misleading as these models may be using different components [8]. Further, the inter-model distance (the distance between projections of two models) was used to estimate the independency between land surface models [14]. Again, this is questionable for some specific cases. For example, if two models are having less inter-model distance and are distinctly spread around the observation, they might still be independent [15]. To overcome the impact of observations, the model performance or model error, i.e., the distance of the model from the observation is estimated by considering the observation data [16]. However, this approach fails to capture the nonlinear dependency among the models, and also to satisfy the equitability property of the dependence measure (giving a similar score to an equally strong relationship irrespective of the relationship being linear, exponential, or periodic) [17,18]. Therefore, in addition to combining the inter-model distance and model performance error, it is essential to derive a methodology that exhibits equitability property, and accounts for both linear and nonlinear dependence between climate models.

## Methodology proposed

In this study, a mutual information-based dependence approach is developed to measure the extent of dependency among GCMs. Mutual information (MI) is a statistical measure that can be used to estimate the mutual dependence between two variables. It is a measure of inherent dependence expressed in the joint distribution of two random variables relative to their marginal distribution under the assumption of independence [19]. MI can help to reduce uncertainty about one GCM given the knowledge of another (See Figure S1) [20]. Therefore, MI between two GCMs is zero, if and only if they are independent GCMs [17,21]. Since mutual information is nonparametric (as it does not assume any sort of underlying distribution or mathematical form of dependence), it can capture both linear and nonlinear dependency between two random variables [19,22]. It is widely accepted that this statistical metric can measure the dependencies without biasness for any kind of relationship [22]. This statistical measure of dependence holds the equitable property as well [18,[23], [24], [25]]. This helps in strengthening the power to measure dependence and allows to capture all types of relationships of a certain minimal strength [18,26,27].

## Measure of dependence by using MI

The model error (e) in the variable ‘*v’* from each GCM ($v^{model}\;$i.e., averaging the model data from the historical period) is calculated as its deviation from the reference observed data ($v^{obs}\;i.e.,\;$averaging the observational data from the historical period), as shown in Eq. (1).

(1) $$e_{x}=v^{obs}-v_{x}^{model}$$

where $e_{x}$ and $v_{x}^{model}$ are the values of error and variable ‘*v’* for *x* model. Here, *x* varies from 1 to *m*, where ‘m’ is the total number of models. For all the models, error term is calculated using the Eq. (1) and the mutual information between every possible pair of model error terms is estimated.

The dependency between two models is estimated as the mutual information between the error of two models. Hence, both model performance (by consideration of model error) and inter-model distance (by consideration of dependence between two model errors) can be combined in the approach.

**Fig. 1:**
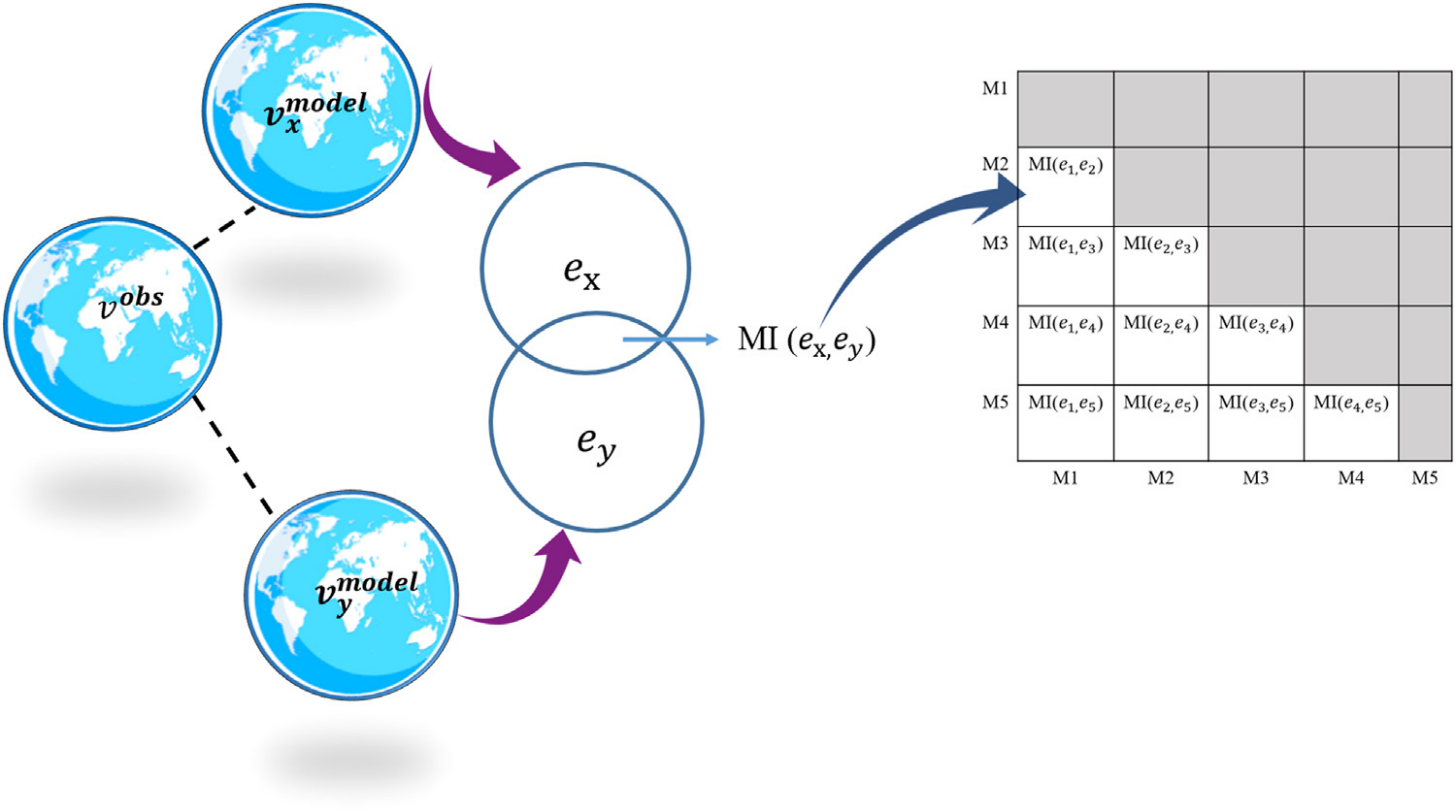
Estimation of mutual information between GCMs (MI shown for every possible pair among five GCMs as an example).

As an illustration, a case including five GCMs is presented in Fig. 1, as an example, for the basic understanding the methodology used. If $v_{x}^{model}$ and $v_{y}^{model}$ are the data from ‘*x’* and ‘*y’* model, then $e_{x,}\;\text{and}\;e_{y}$ are their respective errors/bias from the observed value $v^{obs}$. $MI(e_{x,}e_{y})$ is calculated for every possible pair (as shown in Fig. 1 for five models) as shown in the example, for every pair of models, using Eq. (2),

(2) $$MI\left(e_{x,}e_{y}\right)=\sum_{x=1}^{n}\sum_{y=1,y\neq x}^{n}p\left(e_{x,}e_{y}\right)\;\log\left[\frac{p\left(e_{x,}e_{y}\right)}{p\left(e_{x}\right)p\left(e_{y}\right)}\right]$$

where, $p(e_{x,}e_{y})$ = joint probability mass function between errors of model *x* and *y*, and $p\left(e_{x}\right)\;\text{and}\;p\left(e_{y}\right)$ are marginal probability mass functions of errors of models *x* and *y* respectively.

MI is zero when $e_{x,}$ and $e_{y}$ are statistically independent, i.e., $p(e_{x,}e_{y})$ = $p\left(e_{x}\right)p\left(e_{y}\right)$.

The detail explanation regarding the computation of mutual information can be found from the supplementary material. After getting the dependence of a GCM with remaining GCMs (e.g., *MI*($e_{1,}e_{2}$), *MI*($e_{1,}e_{3}$), *MI*($e_{1,}e_{4}$), *MI*($e_{1,}e_{5}$) for 1st model (M1), and *MI*($e_{2,}e_{1}$), *MI*($e_{2,}e_{3}$), *MI*($e_{2,}e_{4}$), *MI*($e_{2,}e_{5}$) for 2nd model (M2)), weights are assigned to all the models considered as described below. The value of mutual information is more open ended and can range from ‘0′ to ‘∞’ based on complete independence to complete dependence. In order to provide a defined range for the dependency measurement, we used a weighting scheme being motivated by Sanderson et al. [28] and Knutti et al. [12].

## Similitude of a pair of GCMs

The degree of resemblance of a model pair (*x* and *y* models), is estimated as similitude score, using Eq. (3).

(3) $$S_{xy}=e^{-\left(\frac{1}{MI{\left(e_{x,}e_{y}\right)}^{2}}\right)}$$

where $S_{xy}$ represents the similitude score between *x* and *y* model.

If *x* and *y* are independent model, then *MI* is zero and the similitude score $S_{xy}$ will be zero.

## Mutual dependency of a GCM

By aggregating the similitude of a GCM with other GCMs, the mutual dependency of a model with others is determined, as shown in Eq. (4).

(4) $$\;D_{x}=1+\sum_{y=1,y\neq x}^{n}S_{xy}$$

where $D_{x}$ value represents the dependency score of ‘*x’* model in a multi-model ensemble.

Larger the values of $D_{x}$, the more is the dependency of a model with others.

## GCM independence

Once, the dependency of all the models is determined**,** independence weight (W) of each model is calculated as the inverse of its dependency score. The independence weight is estimated using the formula shown in Eq. (5).

(5) $$W_{x}=\frac{1}{D_{x}}$$

where $W_{x}$ is independence weight of ‘*x*’ model.

If the $D_{x}$ will be less, then $W_{x}$ will be larger, which indicates that the model is more independent with more independence weightage.

Further, the weights of each model are normalized such that $\sum_{x=1}^{n}W_{x}=1$.

The proposed methodology is applied for the 3-hourly precipitation projections $\left(v^{model}\right)$ from 20 GCMs of Coupled Model Intercomparison Project 5 (CMIP5) multi-model ensemble. Additionally, precipitation from Japanese 55-year Reanalysis data (JRA-55, Kobayashi et al. [29]) is taken as the reference data $\left(v^{obs}\right)$, which is downloaded from ‘https://climatedataguide.ucar.edu/climate-data/jra-55′.”

**Fig. 2:**
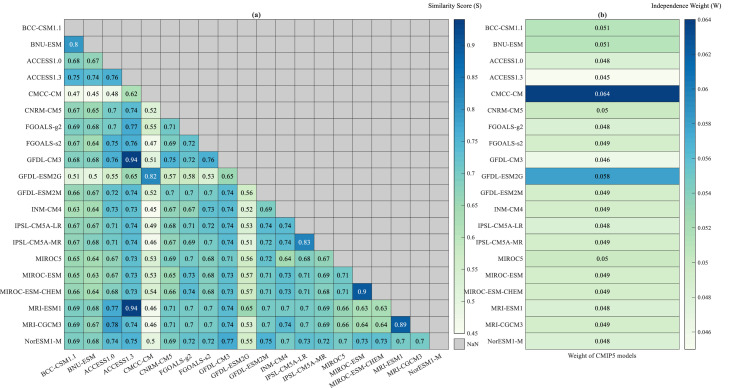
(a) Similitude score of each possible model pair in the CMIP5 models, and (b) Independence weight derived for each model considered in the study.

The similitude of pair of CMIP5 models as depicted in Fig. 2a, varies from 0.45 and 0.94. The CMCC—CM model and GFDL-ESM2G models have significantly lower mutual dependency with remaining models in the ensemble. These two models may have distinct spatial biases as compared to other models due to unique numerical schemes, parameterization processes, representations etc. Therefore, these models with low dependence are assigned higher independence weights as compared to other CMIP5 models. Whereas ACCESS1.3 model has higher similitude and is assigned lower independence weightage (See Fig. 2b) in the ensemble. In case of no dependence among GCMs in the multi-model ensemble, a weightage of 1/20 (0.05) is expected for each model. However, the independence weights vary from 0.045 to 0.064, which implies that the models are inter-dependent. Following the similitude of GCMs, a relatively high independence weight of 0.064 is obtained for CMCC-CM models. The CMCC-CM is observed to be as the most independent GCM. It may be due to its unique resolution (0.7º × 0.7º) and being the only model from the institute “Centro Euro-Mediterraneo per I Cambiamenti Climatici (Italy)”. The model includes ECHAM5 as its atmospheric component, where an implicit scheme for coupling the land surface and the atmosphere, a new scheme for the representation of subgrid-scale orographic effects, and parameterization of convection based on the mass flux concept are incorporated [[7], [8], [9],^30^].

Expectedly, MIROC-ESM and MIROC-ESM-CHEM, which are from same institute and with similar spatial resolution, have very similar independence weightage of 0.049, respectively. While MIROC5 model is also from the same institute, it gives a different independence weight, possibly due to significantly different spatial resolution (Table S1). Similar behavior is seen for other models in the ensemble, as is evident from Fig. 2.

## Weighted variance approach for uncertainty estimation

The weights estimated as per the above methodology, can be used for quantifying the uncertainty among GCMs using the equation below (Eq. 6).

(6) $$\text{Model}\;\text{uncertainty}={\left[\frac{W_{x}{\left(v_{x}-\bar{v}\right)}^{2}+W_{x+1}{\left(v_{x+1}-\bar{v}\right)}^{2}+\cdot \cdot \cdot \cdot \cdot \cdot +W_{m}{\left(v_{m}-\bar{v}\right)}^{2}}{\left(W_{x}+W_{x+1}+\cdot \cdot \cdot +W_{m}\right)}\right]}^{\frac{1}{2}}$$

where *W =* independence weightage of each model

$v_{x}$ = variable ‘*v’* from model ‘*x*’

$\bar{v}$*=* Average value of variable ‘*v’* from *m* models

*x =* model number varying from 1 to m

*m=* total number of models

To investigate the effectiveness of the proposed methodology, uncertainty is quantified in one sub daily precipitation extreme index by considering/ignoring the intermodel dependency. The weighted variance approach resulted reduced uncertainty as compared to the unweighted variance approach (See Figure S2). Additionally, by utilizing this methodology, uncertainty in quantified in the projections of sub-daily precipitation extremes [31].

## Conclusions

While model uncertainty has been studied for a long time, the focus on contributing towards realistic quantification of uncertainty is very limited. Despite substantial improvement in climate modeling over the past few decades, only few modeling groups can afford to develop all the model components uniquely without replicating model code, literature, ideas of representation processes, parameterization schemes of others. The extent of such replication/dependency by all the models needs to be considered for estimating uncertainty in a multi-model ensemble. The suggested method resulted comparatively higher independence weights for the models having unique resolution, modeling center and parametrization schemes etc., and lower independence weights for the models having similarity with others. Therefore, the proposed methodology can do the needful for quantifying uncertainty in a more realistic way by assigning weights to all the GCMs based on their individual dependency with the remaining models.

## Declaration of Competing Interest

The authors declare that they have no known competing financial interests or personal relationships that could have appeared to influence the work reported in this paper.

## Data Availability

Data is made available freely CMIP5/CMIP6.

## Appendix Supplementary materials

Image, application 1
