# Supplementary material for: Mutual information based weighted variance approach for uncertainty quantification of climate projections
Source: MethodsX. 2023 Feb 5;10:102063. doi: 10.1016/j.mex.2023.102063 (PMC9958507; doi:10.1016/j.mex.2023.102063)
Supplement: Supplementary file 1 [file mmc1.docx]

## Supplementary file

***for***

## Mutual Information Based Weighted Variance Approach for Uncertainty Quantification of Climate Projections

**Archana Majhi**, Research Scholar, Department of Civil Engineering, Indian Institute of Technology Delhi, Hauz Khas, New Delhi, India.

**C.T. Dhanya***, Associate Professor, Department of Civil Engineering, Indian Institute of Technology Delhi, Hauz Khas, New Delhi, India.

**Sumedha Chakma**, Associate Professor, Department of Civil Engineering, Indian Institute of Technology Delhi, Hauz Khas, New Delhi, India.

**Corresponding Author:**

C.T. Dhanya, Associate Professor, Department of Civil Engineering, Indian Institute of Technology Delhi, Hauz Khas, New Delhi, India;

Email: [dhanya@civil.iitd.ac.in](mailto:dhanya@civil.iitd.ac.in);

Office Tel.: +91 11 2659 7328.

1. **Detail explanation for estimation of mutual information :**

The error term *e*_x_ is calculated for each model by estimating its deviation from the reference data. In our case for all the 20 models, the error values (*e_1_, e_2_, …e_20_*) from the reference data ($v^{obs} i.e.,$averaged from the years 1979 - 2005) are calculated.

Let us take an example of a single model ‘*x*’ and considered variable is ‘v’. For this model, the $v_{x}^{model}$ is estimated by averaging data from the years 1979 - 2005). The model error ($\boldsymbol{e}_{\boldsymbol{x}}$) in the variable ‘*v’*  is calculated as its deviation from the reference observed data $v^{obs}$, as shown in Equation 1.

$$\boldsymbol{e}_{\boldsymbol{x}}=v^{obs}-v_{x}^{model} (1)$$

where $\boldsymbol{e}_{\boldsymbol{x}}$ and $v_{x}^{model}$ are the values of error and variable ‘*v’*  for *x* model. Here, *x* varies from 1 to *m*, where ‘m’ is the total number of models. For all the models, error term is calculated using the equation 1 and the mutual information between every possible pair of model error terms is estimated using the equation 2 as mentioned below.

Example : The model *x* has error $\left( \boldsymbol{e}_{\boldsymbol{x}} \right)$ values e*_x1_*, e*_x2_*, e*_x3_*, …., e_xi_ (over n number of global land grids) and similarly the model *y* has error $\left( \boldsymbol{e}_{\boldsymbol{y}} \right)$ values e*_y1_*, e*_y2_*, e*_y3_*, …., e*_yi_*_,_ respectively. Let s(e*_xi_*_,_ e*_yi_*) denote the number of samples with e*_xi_*_,_ and e*_yi_* values, and n is the total number of samples. The joint probability mass p(e*_xi_*_,_ e*_yi_*) is estimated as s(e*_xi,_* e*_yi_*)/n. The marginal probability mass p(e*_xi_* ) and p(e*_yi_*) can be accordingly calculated. Then, the mutual information between $\boldsymbol{e}_{\boldsymbol{x,}}$ and $\boldsymbol{e}_{\boldsymbol{y}}$, $MI\left( \boldsymbol{e}_{\boldsymbol{x,}}\boldsymbol{e}_{\boldsymbol{y}} \right)$ is calculated by

$$MI\left( \boldsymbol{e}_{\boldsymbol{x,}}\boldsymbol{e}_{\boldsymbol{y}} \right)=\sum_{x=1}^{n} \sum_{y=1,y\neq x}^{n} p\left( \boldsymbol{e}_{\boldsymbol{x}\mathbf{,}}\boldsymbol{e}_{\boldsymbol{y}} \right)\log\left[ \frac{p\left( \boldsymbol{e}_{\boldsymbol{x}\mathbf{,}}\boldsymbol{e}_{\boldsymbol{y}} \right)}{p\left( \boldsymbol{e}_{\boldsymbol{x}} \right)p\left( \boldsymbol{e}_{\boldsymbol{y}} \right)} \right] (2)$$

where, $p\left( \boldsymbol{e}_{\boldsymbol{x}\mathbf{,}}\boldsymbol{e}_{\boldsymbol{y}} \right)$ = joint probability mass function between errors of model *x* and *y*, and $p\left( \boldsymbol{e}_{\boldsymbol{x}} \right)\mathrm{and}p\left( \boldsymbol{e}_{\boldsymbol{y}} \right)$ are marginal probability mass functions of errors of models *x* and *y* respectively.

1. **Effectiveness of the proposed methodology :**

The figure shows the mutual information between x and y. The orange portion of the circle indicates the amount information shared between x and y which is called as mutual information. Given this mutual information, it reduces the uncertainty about x by knowing y and vice versa. The reduced uncertainty left about x is denoted by blue portion of the circle.

**
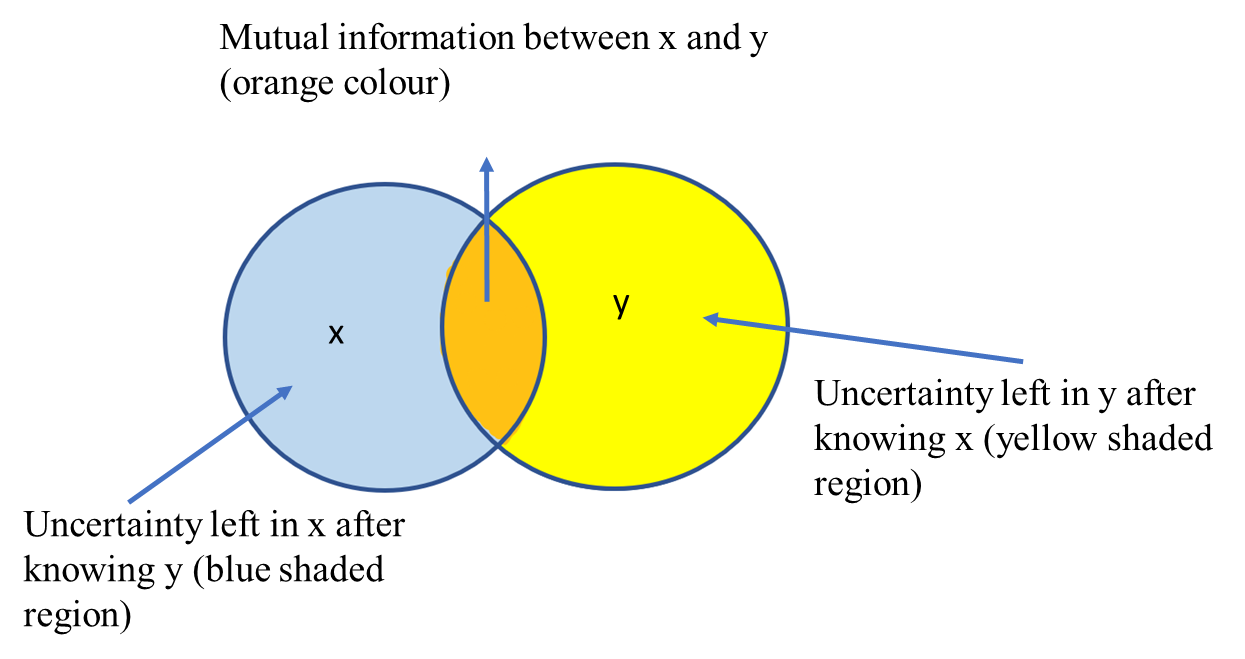
**

**Figure S1. The mutual information between x and y illustrating the reduction of uncertainty**

To investigate the effectiveness of the proposed methodology, as an example, uncertainty is quantified in one sub daily precipitation extreme index (eg., maximum wet spell length) by both considering and ignoring the interdependency. From the analysis, it has been noticed that the models showed uncertainty range of 0-18 hours when inter-dependency among models is considered. When we analyzed the uncertainty ignoring the interdependency, the uncertainty is observed to be of higher range (> 70 hours) which is not the actual range since in this case, models are assumed to be independent of each other which is not the real case.

**Figure S2. Reduction of uncertainty in maximum wet spell length (hours) using mutual information**

**Table S1** List of CMIP5 models considered in this study.

| **Model** | **Institution** | **Spatial Resolution (Degrees)** |
| --- | --- | --- |
| ACCESS1.0 | Commonwealth Scientific and Industrial Research Organisation (CSIRO) - Bureau of Meteorology (BOM), Australia | 1.9 × 1.2 |
| ACCESS1.3 | BOM, Australia | 1.9 × 1.2 |
| BCC-CSM1.1 | Beijing Climate Center, China Meteorological  Administration | 2.8 × 2.8 |
| BNU-ESM | College of Global Change and Earth System  Science, Beijing Normal University | 2.8 × 2.8 |
| CMCC-CM | Centro Euro-Mediterraneo per I Cambiamenti Climatici (CMCC), Italy | 0.7 × 0.7 |
| CNRM-CM5 | Centre National de Recherches Météorologiques | 1.4 × 1.4 |
| FGOALS-g2 | State Key Laboratory of Numerical Modeling for Atmospheric Sciences and Geophysical Fluid Dynamics (LASG)–Center for Earth System Science (CESS), China | 2.8 × 2.8 |
| FGOALS-s2 | LASG–Institute of Atmospheric Physics (IAP), China | 1.6 × 2.8 |
| GFDL-CM3 | NOAA Geophysical Fluid Dynamics Laboratory | 2 × 2.5 |
| GFDL-ESM2G | NOAA Geophysical Fluid Dynamics Laboratory | 2.0 × 2 |
| GFDL-ESM2M | NOAA Geophysical Fluid Dynamics Laboratory | 2.0 × 2.5 |
| INM-CM4 | Institute of Numerical Mathematics (INM), Russia | 1.5 × 2 |
| IPSL-CM5A-LR | Institut Pierre-Simon Laplace | 1.9 × 3.8 |
| IPSL-CM5A-MR | Institut Pierre-Simon Laplace | 1.3 × 2.5 |
| MIROC5 | Atmosphere and Ocean Research Institute (The University of Tokyo), National Institute for Environmental Studies, and Japan Agency for Marine-Earth Science and Technology | 1.4 × 1.4 |
| MIROC-ESM | Japan Agency for Marine-Earth Science and Technology, Atmosphere and Ocean Research Institute (The University of Tokyo), and National Institute for Environmental Studies | 2.7 × 2.8 |
| MIROC-ESM-CHEM | Japan Agency for Marine-Earth Science and Technology, Atmosphere and Ocean Research Institute (The University of Tokyo), and National Institute for Environmental Studies | 2.8 × 2.8 |
| MRI-ESM1 | Meteorological Research Institute | 1.1 × 1.1 |
| MRI-CGCM3 | Meteorological Research Institute | 1.1 × 1.1 |
| NorESM1-M | Norwegian Climate Centre | 1.1 × 1.1 |
